# Radiation-Induced Modifications in Bovine Serum Albumin in Saline Solutions Under E-Beam Irradiation

**DOI:** 10.3390/ijms27135952

**Published:** 2026-07-02

**Authors:** Victoria Ipatova, Ulyana Bliznyuk, Polina Borshchegovskaya, Arkady Braun, Alexander Chernyaev, Maria Toropygina, Alexander Nikitchenko, Anastasia Oprunenko, Aleksandr Kozlov, Iana Zubritskaya, Igor Rodin, Elena Kozlova

**Affiliations:** 1 Skobeltsyn Institute of Nuclear Physics, Lomonosov Moscow State University, GSP-1, 1-2 Leninskiye Gory, 119991 Moscow, Russia; ipatova.vs15@physics.msu.ru (V.I.); alexeevapo@mail.ru (P.B.); a.p.chernyaev@yandex.ru (A.C.); zubritckaia.iv18@physics.msu.ru (I.Z.); 2 Laboratory of Digital Radiation Technologies, Central University, Gasheka Street, 7, 123056 Moscow, Russia; nikitchenko.ad15@physics.msu.ru; 3 Department of Physics, Lomonosov Moscow State University, GSP-1, 1-2 Leninskiye Gory, 119991 Moscow, Russia; 4 Department of Chemistry, Lomonosov Moscow State University, GSP-1, 1-3 Leninskiye Gory, 119991 Moscow, Russia; avbraun@yandex.ru (A.B.); oprunenko_anastasiya@mail.ru (A.O.); igorrodin@yandex.ru (I.R.); 5 Department of Medical and Biological Physics, Sechenov First Moscow State Medical University, 8-2 Trubetskaya Str., 119991 Moscow, Russia; tim.mmit@yandex.ru (M.T.); fillnoise@mail.ru (A.K.); waterlake@mail.ru (E.K.); 6 Department of Analytical Chemistry, Lomonosov Institute of Fine Chemical Technologies, Moscow State Institute of Radiotechnics, Electronics and Automation—Russian Technological University (MIREA—RTU), 78 Vernadsky Avenue, 119571 Moscow, Russia; 7 Department of Epidemiology and Evidence-Based Medicine, Sechenov First Moscow State Medical University, 8-2 Trubetskaya Str., 119991 Moscow, Russia

**Keywords:** protein, bovine serum albumin (BSA), electron beam irradiation, reactive oxygen species, protein modification, radiation-induced protein damage, dose–concentration model, food irradiation

## Abstract

Electron beam irradiation, extensively used for suppressing a wide range of pathogens contaminating food products, pharmaceuticals and biological raw materials, inevitably damages the surrounding proteins, stripping the product of its essential nutritional and functional properties. This issue can be addressed by adjusting the electron beam irradiation dose, bearing in mind the concentration of proteins in the product since it can affect the rate of radiation-induced modifications in proteins. The study investigates the impact of 7.5 MeV electron-beam irradiation on modifications in bovine serum albumin (BSA) molecules in 0.9% NaCl solution in the concentration range of 0.5–70 mg/mL, encompassing a wide range of protein concentrations in food products, pharmaceuticals and biological raw materials. Conformational changes and aggregation of BSA were evaluated using UV–Vis spectrophotometry at λ = 350 nm. Peptide bond rupture in protein native structures was assessed by performing HPLC-MS/MS analysis after trypsin hydrolysis using three selected peptides located in different domains of the BSA amino acid sequence. It was found that the rate of radiation-induced modifications increased with an increase in the irradiation dose but decreased markedly as BSA concentration increased. While at the BSA concentration of 0.5 mg/mL over 87% of BSA molecules underwent peptide bond rupture under irradiation with a dose of 5 kGy, a two-fold increase in the BSA concentration and irradiation dose enabled bond rupture in only 20% of BSA molecules. Our experimental approach resulting in the development of the dose and concentration model allows us to quantify the degree of radiation-induced protein modifications depending on the irradiation dose and protein concentration in food products, pharmaceuticals and biological raw materials.

## 1. Introduction

Over the past few years, irradiation technologies have spanned a wide range of industries including the food industry, medicine and pharmaceuticals and are increasingly used to reduce microbial load in food products and biological raw materials [1,2,3] and enhance the bioavailability of biopolymers and protein-based pharmaceuticals [4]. Most industrial irradiation facilities use electron accelerators since they can control the penetration depth of electrons by varying the operating mode and energy of the electrons, which allows for greater flexibility in treating different layers of the product depending on the irradiation goal [5,6,7,8]. Electron beam irradiation with 10 MeV electrons is predominantly used in irradiation of food products and materials; the recent recommendations of the IAEA include the use of low-energy electrons and photons in industrial irradiation facilities since these irradiation sources can be easily integrated into the production process and are safer to use [9]. Low-energy irradiation is efficient for surface treatment of fruit and vegetables and food categories which are prone to contamination of surface layers. The use of 1–3 MeV electron accelerators can also be beneficial for irradiation crosslinking of polymers in order to treat heat-shrink materials [10,11,12].

Electron beam irradiation is the method of choice at irradiation facilities since it suppresses a wide range of pathogens contaminating food products and biological raw materials. However, the downside of irradiation is that it damages the surrounding molecules which are essential for the nutritional and functional properties of the product, determined by proteins which are the active components of many pharmaceuticals, such as therapeutic drugs, enzyme systems, vaccines, serum preparations, antibodies, and transport proteins [13,14,15]. Thus, it is important to study the impact of electron beam irradiation on the functional, structural and biochemical properties of proteins in food matrices and pharmaceuticals.

The functional properties of proteins are directly determined by their spatial organization, supported by a system of weak non-covalent interactions, such as hydrogen bonds, hydrophobic interactions, electrostatic forces, and disulfide bonds [16]. Irradiation induces structural modifications in proteins; this is especially critical for aquatic systems, where proteins are in a hydrated state and become sensitive to water radiolysis products, causing the disruption of the proteins’ native structure, leading to functional changes, including to their solubility, bioavailability, enzymatic activity, transport properties and tendency to aggregate [17,18,19,20]. Since the suppression of microorganisms in food products and protein-based pharmaceuticals using irradiation cannot occur without a negative impact on proteins, it is highly important to the determine optimal irradiation doses that can meet the irradiation goal without a detrimental effect on proteins [21,22,23].

Under irradiation, proteins in water-containing food products and pharmaceuticals are damaged due to interaction with water radiolysis products and reactive oxygen species (ROS) [24,25]. Being the main targets of ROS, such as hydroxyl radicals (•OH), superoxide anion radicals, hydrogen peroxide, and other highly reactive particles capable of initiating oxidative processes, proteins undergo modification of amino acid side chains, disruption of hydrophobic interactions, changes in the tertiary structure, and formation of aggregates [25,26,27]. Considering that the degree of protein damage is determined by a wide range of factors, including water content [24,25], pH [28], oxygen concentration [29,30], antioxidants [18], and the concentration of the protein itself, optimal irradiation doses should factor in the individual properties of food products and pharmaceuticals that have unique protein compositions and distributions of protein concentrations.

Being one of the most thoroughly studied proteins, bovine serum albumin (BSA) is a suitable substance for investigating radiation-induced changes in proteins under in vitro conditions due to its high solubility, stability, and sensitivity to external influences [18,28,29,30]. The study chooses BSA as a protein for studying radiation-induced modifications since BSA serves as a highly versatile globular protein in the pharmaceutical industry, biotechnology, and the food industry where e-beam irradiation has become the method of choice for its versatility, reliability and high processing speed.

Most recent studies consider the effect of irradiation dose without taking into account protein concentration and the distribution of radiation-chemical processes in the aquatic environment [19,31,32,33]. However, it is the protein concentration that determines the likelihood of reactive oxygen species interacting with a single target molecule and it can significantly affect the rate of radiation-induced changes in protein molecules [34]. Since food products and pharmaceuticals contain a wide range of concentrations of proteins that can influence the degree of radiation-induced changes in protein molecules, studying the impact of protein concentration on protein damage within the optimal irradiation dose range has become a priority for enhancing irradiation efficiency. The aim of the study is to assess the impact of 7.5 MeV electron beam irradiation on radiation-induced modifications in BSA molecules in aquatic solutions with different BSA concentrations using spectrophotometry and liquid chromatography as they supplement each other since they allow the investigation of both spatial modifications and chemical bond rupture in the BSA molecule.

## 2. Results

### 2.1. Research Stages

**Figure 1:**
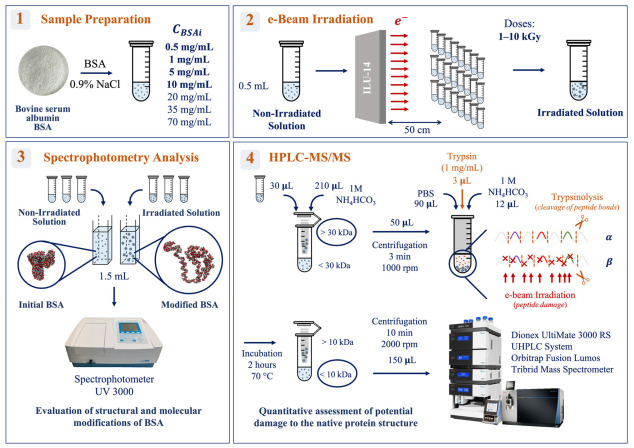
Stages of experimental study: (**1**) preparation of the BSA solutions at different concentrations; (**2**) electron beam irradiation of solutions; (**3**) spectrophotometry analysis; (**4**) HPLC-MS/MS analysis. Red arrows indicate the direction of the e-beam irradiation. Orange dashed lines represent the trypsin cleavage sites in the BSA amino acid sequence, red crosses denote peptide bonds damaged by e-beam irradiation.

The work investigated structural modifications in BSA in water solution after exposure to 7.5 MeV accelerated electrons with different doses ranging from D_1_ = 1 kGy to D_10_ = 10 kGy. Figure 1 shows the four stages of the study, with a detailed description of each stage given in Section 4.

At the first stage, bovine serum albumin (BSA) was dissolved in 0.9% NaCl solution at concentrations ranging from 0.5 to 70 mg/mL to investigate the dose and concentration dependencies of the rate of BSA modifications during electron beam irradiation. At the second stage, BSA solutions at different concentrations were irradiated with accelerated electrons with the doses of 1, 2, 3, 5, 7, and 10 kGy using the ILU-14 pulsed electron accelerator. At stage three and stage four, non-irradiated and irradiated solutions were analyzed using spectrophotometry and high-performance liquid chromatography coupled with high-resolution mass spectrometry (HPLC-MS/MS). The structural and molecular changes in bovine serum albumin were evaluated by detecting the changes in the optical absorption spectra of the BSA solutions to quantify the rate of structural modifications. To determine the rate of the damage to the protein native structure, the concentrations of three selected peptides from three domains of BSA amino acid sequence were measured after trypsinolysis was performed upon irradiation in order to obtain dose dependencies of the oxidation rate of chemical bond rupture in BSA molecules in water solutions at different BSA concentrations. After determining dose–concentration dependencies for radiation-induced BSA modifications we developed a mathematical model which predicts the rate of the damage to the proteins, which are contained in different concentrations in food products, pharmaceuticals and other biological objects, in order to find the optimal irradiation parameters and increase irradiation efficiency.

### 2.2. Absorption Spectra of Solutions at Different BSA Concentrations

**Figure 2:**
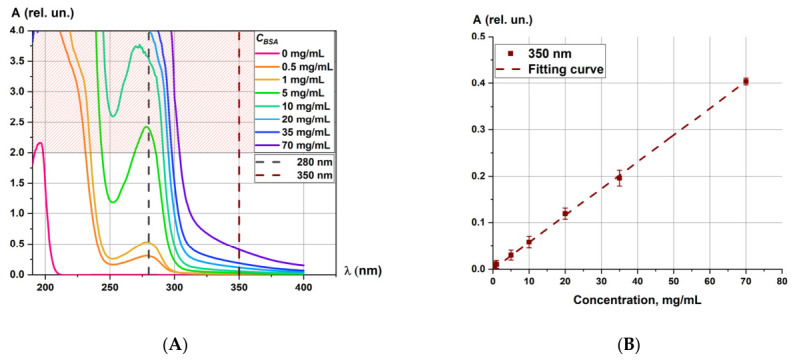
(**A**) Absorption spectra of BSA solutions in the concentration range of 0.5–70 mg/mL. The black and brown dotted lines mark the wavelengths λ = 280 nm and 350 nm, respectively. The red shaded area denotes the non-operating wavelength range of the spectrophotometer. (**B**) The dependency of the optical density of the BSA solutions on the BSA concentration measured at the wavelength of λ = 350 nm.

Before estimating the dose dependencies of structural modifications in BSA molecules in water solutions at different concentrations after irradiation, we performed model experiments involving the measurement of UV and visual absorption spectra of non-irradiated BSA solutions at different BSA concentrations ranging from 0.5 to 70 mg/mL. The concentration range was chosen to cover a wide range of protein content in real food products, such as milk and dairy products, meat and meat extracts, protein-containing foods of animal and plant origin, as well as protein-based pharmaceuticals [35,36,37,38,39]. The absorption spectra of the BSA solutions were studied in the wavelength range of 190–400 nm, since this range is suitable for measurement of optical scattering of BSA molecules which can be used as a non-destructive technique to analyze the protein structure, size, aggregation, and interactions of BSA molecules in water solutions [33,40]. Figure 2A shows the absorption spectra of the BSA solutions at the concentration range of 0.5–70 mg/mL.

With an increase in the BSA concentration from 0.5 mg/mL to 70 mg/mL, an increase in the absorption and scattering spectra is observed in the wavelength range of 190–400 nm (Figure 2A). As it can be seen from Figure 2A, the BSA absorption spectra demonstrates a complex behavior in the region of 250–300 nm due to the light absorption of aromatic amino acids: tryptophan (80%), tyrosine and phenylalanine [37]. The maximum in the absorption spectra at λ ≈ 280 nm is commonly used to assess protein structure and concentration [33,40,41,42]. However, at high BSA concentrations above 5 mg/mL, the optical density at the given wavelength exceeds the operating range of the spectrophotometer, which leads to an off-scale effect and a violation of the linearity of the response. Further analysis was carried out at λ = 350 nm, where the intrinsic electron absorption of BSA is negligible and the optical density is formed mainly due to light scattering. As can be seen from Figure 2B, the optical density at λ = 350 nm demonstrates a linear dependency on the BSA concentration in the range from 0.5 to 70 mg/mL:

(1) $$A_{sct}=kC,$$

where k = 0.00577 ± 0.00006 (mg/mL)^−1^ is a linear coefficient and C (mg/mL) is the BSA concentration in the solution. The linear dependency of the optical density of the BSA solution measured at λ = 350 nm on the BSA concentration determined the choice of the working wavelength which is used to estimate the ratio of radiation-induced modified BSA molecules in saline solutions after irradiation with different doses.

### 2.3. Absorption Spectra of Irradiated BSA Solutions at Different Initial Concentrations

#### 2.3.1. Spectrophotometry Method

**Figure 3:**
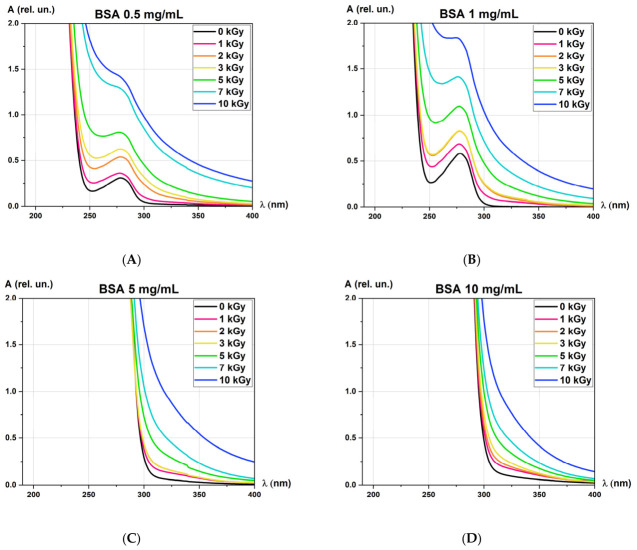
Spectra of BSA solutions after electron beam irradiation at different initial concentrations: (**A**) 0.5 mg/mL, (**B**) 1 mg/mL, (**C**) 5 mg/mL, and (**D**) 10 mg/mL, λ = 190–400 nm.

The absorption and scattering spectra of BSA solutions at the initial concentrations of 0.5–10 mg/mL after irradiation with 7.5 MeV accelerated electrons with the doses ranging from 1 to 10 kGy are shown in Figure 3A–D.

An increase in the irradiation dose caused the optical density in the short-wavelength region of the spectra ranging from 190 to 300 nm to increase, which prompted the formation of a pronounced long-wavelength tail at λ > 300 nm due to the appearance of radiation-induced modified BSA molecules and protein aggregates. The results of our study correspond to the findings of other recent studies since the formation of larger protein aggregates contributes to a higher scattering rate [19,32,43].

**Figure 4:**
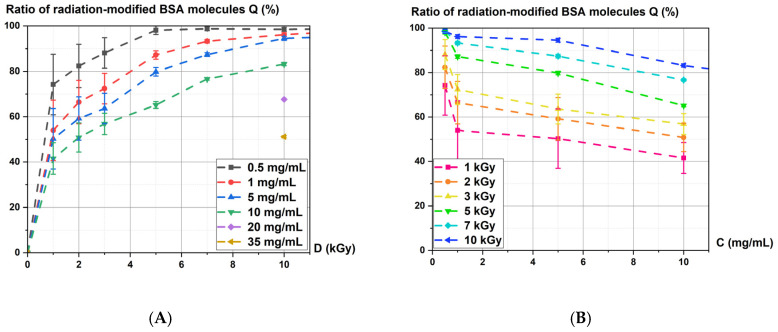
(**A**) The dose dependency Q(D) of the ratio of radiation-induced modified BSA molecules at different concentrations of BSA and (**B**) the concentration dependency Q(C) of the ratio of radiation-induced modified BSA molecules occurring as a result of irradiation with different doses.

The dose dependency Q(D) of the ratio of radiation-induced modified BSA molecules at different BSA concentrations and the concentration dependency Q(C) of the ratio of radiation-induced modified BSA molecules occurring as a result of irradiation with different doses are shown in Figure 4A,B, calculated using Formula (10).

For all the BSA concentrations considered in this research, a monotonous nonlinear increase in the ratio of the modified proteins Q was observed with an increase in the irradiation dose (Figure 4A). As can be seen from Figure 4A, a decrease in the BSA concentration led to an increase in the rate of accumulation of modified proteins Q as the irradiation dose increased. The lower the BSA concentration, the lower the dose at which Q(D) dependency reached a plateau, indicating that the level of the modified BSA molecules reached 85–95%.

The concentration dependency Q(C) of the ratio of radiation-induced modified BSA molecules shows that the proportion of the modified proteins decreases with an increase in the concentration in the entire dose range of 1–10 kGy (Figure 4B). The most pronounced decrease in the rate of modified protein accumulation is observed at concentrations up to 2–3 mg/mL, after which the dependency Q(C) becomes more gradual, indicating a nonlinear effect of the concentration on the radiation-induced modified BSA molecules irradiated with the same dose.

#### 2.3.2. HPLC-MS/MS Analysis

**Figure 5:**
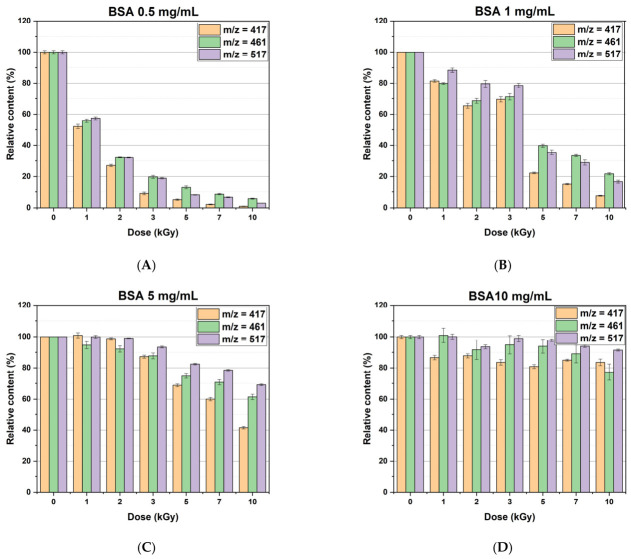
Relative concentration diagrams for the selected peptides T35–44 (*m*/*z* = 417), T249–256 (*m*/*z* = 461), T548–557 (*m*/*z* = 517) after 7.5 MeV e-beam irradiation and trypsinolysis at the initial concentrations: (**A**) 0.5 mg/mL, (**B**) 1 mg/mL, (**C**) 5 mg/mL, and (**D**) 10 mg/mL.

The dependency of the concentration of three selected peptides T35–44, T249–256, T548–557 from three domains of the amino acid sequence of BSA irradiated with 7.5 MeV electrons in the water solutions at the initial concentrations of 0.5–10 mg/mL are shown in Figure 5A–D and in Table A1 in Appendix A.

The experimental data show that with an increase in the dose, the relative concentration of the selected peptides registered in the water solution after trypsinolysis decreased nonlinearly for all concentrations of BSA studied in our research. At the same time, a clear effect is observed: with an increase in the concentration of BSA from 0.5 mg/mL to 10 mg/mL, the rate at which the relative content of the selected peptides decreased at higher irradiation doses slowed down due to a higher number of ROS interacting with BSA molecules.

A clear effect of the BSA concentration on the rate of modified protein accumulation in BSA suspensions irradiated with the same dose observed using spectrophotometry and HPLCMS/MS methods allows to use the revealed dose and concentration dependencies as the basis for a mathematical model in order to quantify the degree of radiation-induced protein modifications depending on the irradiation dose and protein concentration in foods containing proteins and protein-based pharmaceuticals.

### 2.4. Dose and Concentration Dependency Model

**Figure 6:**
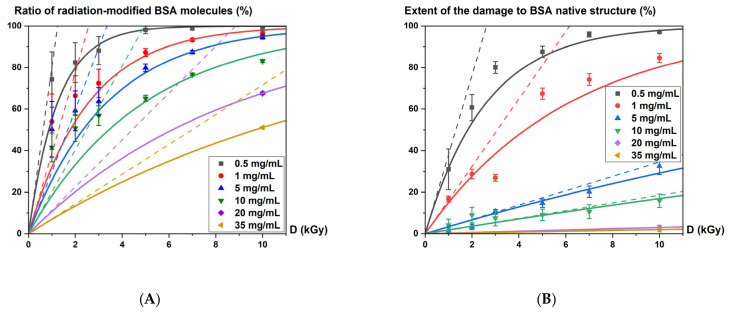
The dose dependency of the ratio of radiation-modified BSA molecules at the initial concentrations of 0.5–10 mg/mL measured using: (**A**) spectrophotometry method (λ = 350 nm); (**B**) HPLC–MS/MS analysis. Symbols represent experimental data; curves show the exponential fit functions (Equation (2)).

Since the selected peptides T35–44, T249–256, and T548–557 from three different BSA domains demonstrate a consistent dose-dependent decrease in the relative concentration with an increase in the irradiation dose for all studied concentrations of BSA, the effect of peptide bond rupture in BSA is represented as the mean value across the three selected peptides. It has been found that the rate of peptide bond rupture in BSA amino acid sequences registered using the HPLC–MS/MS method as well as the rate of structure modifications and the formation of aggregates detected using the spectrophotometry method have a similar dependency on the irradiation dose Q(D) and BSA concentration Q(C) when BSA solutions were irradiated with 7.5 MeV accelerated electrons with a dose range of 0–10 kGy (Figure 6A,B).

As it can be seen from Figure 6A,B, for all the concentrations C ranging from 0.5 to 35 mg/mL, the rate of the damaged BSA molecules and the ratio of the modified BSA detected by light scattering measured at the wavelength of 350 nm can be approximated using exponential dependency on the irradiation dose D:

(2) $$\varepsilon \left(D\right)=\left(1-e^{-kD}\right)\cdot 100\%,$$

where ε (%) is the detected radiation-induced effect, k (Gy^−1^) is the rate of radiation-induced BSA modifications.

The accumulation rates of radiation-induced spatial rearrangements of BSA molecules and peptide bond rupture, which were measured using two different methods, slow down with a higher BSA concentration: the dependencies ε(D) become flatter, and achieving high values of ε requires significantly higher doses (Figure 6A,B). At a low concentration of 0.5 mg/mL, the exponential increase in the detected radiation-induced effect is most pronounced and saturation is already achieved at doses of 3–5 kGy, while at high BSA concentrations the increase in the effect ε occurs more gradually and saturation is expected at the doses exceeding 10 kGy.

**Figure 7:**
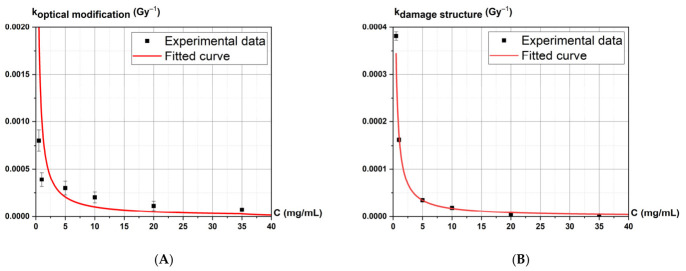
The experimental dependencies k(C) and the fitting curve calculated using Formula (3). (**A**) Spectrophotometry data (λ = 350 nm); (**B**) HPLC–MS/MS data.

Figure 7A,B shows the concentration dependencies k(C) representing the rate of radiation-induced BSA modifications measured using two different methods. As can be seen, the k value decreases hyperbolically with an increase in the BSA concentration; the experimental dependencies k(C) can be approximated using the following formula:

(3) $$k\left(C\right)=\frac{\alpha }{C},$$

where α ((mg/mL)/Gy) is an empirical coefficient characterizing radiation-induced protein damage under fixed irradiation conditions.

The dependencies k(C) reflect the nonlinear effect of the initial BSA concentration on the rate of radiation-induced albumin modifications under e-beam irradiation. At low protein concentrations, a high density of reactive oxygen species in the water solution leads to intense damage to peptide bonds and high k values. As the concentration of BSA increases, the probability of radical interaction per BSA target molecule decreases causing the rate of radiation-induced BSA modifications to decrease.

The hyperbolic dependency k(C) and the exponential dependency ε(D) found using spectrophotometry and the HPLC-MS/MS method are expressed as a single function taking into account the dependency of the radiation effect ε on both the dose D and concentration C:

(4) $$\varepsilon \left(C,D\right)=(1-exp\left(-\frac{\alpha D}{C}\right))\cdot 100\%,$$

where ε(C,D) (%) is a predicted radiation effect on the BSA molecules after irradiation with the dose D (Gy) at the initial protein concentration C (mg/mL); α ((mg/mL)/Gy) is the probability of BSA modification as a result of a single ionization event.

**Figure 8:**
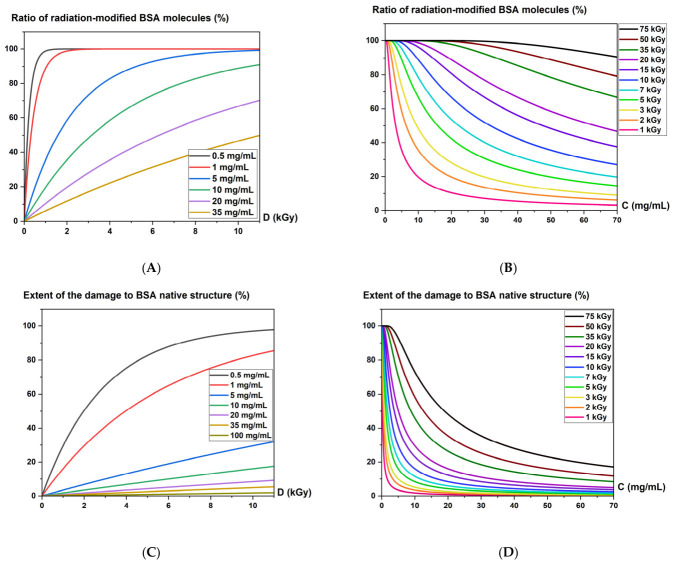
The dose–concentration dependencies ε(C,D) estimated using formula (4). The dependency of the ratio of the BSA modifications detected using the spectrophotometry method on the irradiation dose for different initial concentrations 0.5–100 mg/mL (**A**) and on the protein concentration at the fixed absorbed doses 1–75 kGy. (**B**) The dose dependency of the ratio of BSA with a damaged native structure determined by HPLC–MS/MS on the irradiation dose for different initial concentrations (0.5–100 mg/mL) (**C**) and on the protein concentration at fixed absorbed doses 1–75 kGy (**D**).

The calculated dependencies (4) of the ratio of the BSA spatial modifications revealed by light scattering and the ratio of the BSA molecules with the damaged native structure estimated using the concentration of three selected peptides for different initial protein concentrations ranging from 0.5 to 100 mg/mL and different e-beam doses ranging from 1 to 75 kGy are shown in Figure 8. The approximation curves (4) are in agreement with experimental data, confirming the adequacy of the proposed dose–concentration model with a correlation coefficient of more than 0.9.

The calculated dose–concentration dependencies ε(C,D) reproduced the main experimental trend observed for both analytical methods: at low BSA concentrations, the fraction of modified protein molecules rapidly approached 100%, whereas increasing protein concentration progressively reduced the radiation-induced response at a fixed dose. At the same time, the HPLC–MS/MS data required substantially higher doses to achieve the same level of effect as that observed by spectrophotometry at identical protein concentrations. This indicates that conformational rearrangements and aggregation of BSA molecules occur at lower doses than the rupture of peptide bonds and the loss of native protein structure.

## 3. Discussion

### 3.1. Comparative Study

Our findings indicate that e-beam irradiation of BSA in saline solutions causes two experimentally distinguishable levels of protein modifications. The optical density of the absorption spectra of a BSA solution measured at λ = 350 nm primarily reflects conformational rearrangements, unfolding, and aggregation through increased light scattering, whereas HPLC–MS/MS after trypsinolysis reflects deeper damage to the native protein structure through the loss of selected markers that are located in different regions of the BSA amino acid sequence and represent different structural domains of the protein.

Our results are consistent with the recent studies on gamma-irradiated BSA solutions, where increased optical density in the region of 260–360 nm was associated with aggregate formation, modification of aromatic amino acid residues, and changes in secondary and tertiary protein conformation [18,19,44]. Similar studies reported increases in BSA particle size from 7–8 to 13–17 nm when BSA solutions were irradiated with doses up to 10 kGy, supporting the assumption that radiation-induced light scattering is related to protein aggregation [19,23]. The authors in related study [44] indicate that structural modifications in BSA molecules detected by measuring the intensity of the absorption peak at the wavelength of 280 nm after gamma irradiation of the 0.4% BSA solution with doses of 1 and 5 kGy are associated with conformational rearrangements in the secondary structure of the protein, which were further confirmed by Fourier-transform infrared spectroscopy of the irradiated BSA solution. In addition, a 1.5-fold decrease in the molecular weight of BSA after irradiation with doses of 1 and 5 kGy was reported, indicating partial degradation and fragmentation of the protein molecule rather than its complete destruction. Overall, these findings support our assumption that irradiation-induced changes in the UV absorption spectra of BSA are associated with a combination of conformational rearrangements, aggregation processes, modification of aromatic amino acid residues, and partial degradation of the protein structure. Similar observations have also been reported for other proteins. Dose-dependent increases in optical density and aggregation were observed for ovalbumin, catalase, and hemoglobin following exposure to γ-rays or accelerated electrons, indicating that conformational destabilization and aggregation are common responses of proteins to ionizing radiation [17,45,46].

Since reactive oxygen species (ROS) generated during water radiolysis as a result of interactions between accelerated electrons and H_2_O molecules react with BSA molecules, irradiation induces oxidation of amino acid residues, conformational rearrangements, aggregation, and partial fragmentation of the protein structure. With increasing BSA concentration, proteins being acceptors of free radicals partially screen each other and reduce the effective diffusion length of ROS in the aqueous phase, which decreases the probability of radical attack on a single protein molecule and results in the observed dose–concentration dependency.

It should be noted that the radiation-chemical yield of ROS is influenced by the concentration of dissolved oxygen in irradiated solutions. Direct measurements under 10 MeV electron irradiation demonstrated dose-dependent oxygen depletion in aqueous and albumin-containing systems [47,48,49], caused by reactions of dissolved oxygen with hydrated electrons and hydrogen radicals. Studies [50,51] on e-beam irradiation of aqueous solutions at kGy-level doses also show that dissolved oxygen availability strongly affects radiolytic pathways and secondary product formation. As the absorbed dose increases, dissolved oxygen is progressively consumed, which changes the balance between oxidative and reductive radical pathways. Oxygen depletion can suppress the formation of peroxyl radicals and hydroperoxides and, therefore, reduce the effective yield of oxygen-dependent secondary oxidative damage. This explains why the maximum accumulation rate of modified BSA molecules was observed at doses below 1 kGy. At higher doses, progressive oxygen depletion limits ROS-mediated oxidation processes and changes the kinetics of protein modification, despite the continued increase in the absorbed dose. Consequently, the dose dependency of BSA modification reflects not only the accumulation of ionization events, but also dynamic changes in the oxygen content and radical chemistry of the irradiated aqueous system.

The HPLC–MS/MS results reveal a second level of radiation-induced damage associated with peptide bond rupture in protein native structure. As the number of ROS interacting with a BSA molecule increases, oxidative modification of amino acid residues is followed by peptide bond rupture and the formation of lower-molecular-weight fragments [44,52,53,54]. Similar observations were reported for irradiated watermelon-seed proteins, where an increase in low-molecular-weight peptides accompanied increasing γ-ray doses from 4.8 to 28.8 kGy [52]. Our findings that lower BSA concentrations are responsible for more intense rupture of the peptide bonds in the BSA molecules irradiated in the water solutions at the same dose align with the research results [53]. Furthermore, mass-spectrometric analysis of irradiated ovalbumin revealed radiation-induced peptide modifications analogous to those detected in the present study [55] suggesting that peptide-level damage is not unique to BSA but represents a general consequence of oxidative protein degradation.

Comparative studies of different proteins have demonstrated substantial differences in radiosensitivity. Ovalbumin, casein, hemoglobin, catalase, and BSA exhibit different rates of structural alteration and fragmentation under irradiation, reflecting differences in amino acid composition, tertiary structure, and accessibility of ROS-sensitive residues. Notably, BSA was shown to be more sensitive to γ-irradiation than casein and bovine hemoglobin [45]. However, previous studies primarily focused on dose effects, whereas the influence of protein concentration remained largely unexplored.

The principal novelty of the present study lies in the demonstration, using two independent analytical methods, that protein concentration is a key parameter governing radiation-induced protein damage in aqueous systems. While the influence of absorbed dose on protein modification has been extensively reported, the role of protein concentration has received considerably less attention despite its direct impact on the number of reactive oxygen species available per target molecule. The proposed dose–concentration model provides a quantitative framework for describing this effect and offers a mechanistic interpretation of the experimentally observed nonlinear transition from nearly complete protein modification at low concentrations to only partial modification at high concentrations.

### 3.2. Mechanistic Interpretation of the Dose–Concentration Dependency Model

As it can be seen from Figure 8B,D, the dose–concentration dependencies ε(C,D) estimated using formula (4) demonstrate similar behavior with regard to the effect of BSA structure modification and peptide bond rupture on the concentration C_BSA_ when BSA solution is irradiated with the dose D: at low C_BSA_, the ratio of the modified BSA molecules is close to 100%, whereas with an increase in protein concentration C_BSA_, a monotonous decrease in the observed effect occurs. Each dose–concentration dependency ε(C,D) has a threshold concentration C_tr_, which can be determined as follows: if C_BSA_ ≤ C_tr_, the ratio of the modified BSA molecules is close to 100%, and if C_BSA_ > C_tr_, the ε value decreases with an increase in the protein concentration C_BSA_. Since the key mechanism behind the damage of organic molecules in water solution is indirect oxidation through ROS, a certain minimum number of ROS interactions with BSA molecules is required to trigger BSA spatial modification or peptide bond rupture. A decrease in the ratio of the modified BSA molecules with a higher BSA concentration exceeding the threshold concentration C_tr_ for all BSA solutions irradiated with different doses indicates that the number of ROS interacting with one BSA molecule is less than N_min_ required for the BSA modification effect to be detected.

It can be noted that with an increase in the irradiation dose the threshold concentration C_tr_ shifts to higher values. With an increase in the irradiation dose D a higher number of ROS are present in the BSA solutions, so the plateau effect for dose–concentration dependency ε(C,D), which indicates that all the BSA molecules present in water solution undergo at least N_min_ ROS interactions required for the BSA modification effect to be detected, can be observed at a higher concentration of C_BSA_.

**Figure 9:**
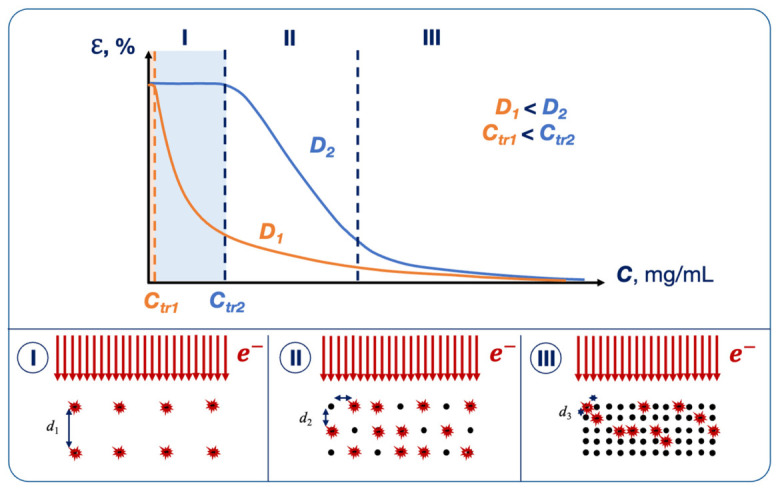
Three distinct ranges of radiation-induced protein concentration effect on BSA damage in saline solution irradiated with the dose D. Regions I–III correspond to different ratios between the BSA concentration (C_BSA_) and the number of radiation-induced ionization events (αD): (**I**) C_BSA_ ≪ αD, where nearly all BSA molecules are modified and ε → 100%; (**II**) C_BSA_ > αD and C_BSA_ > C_tr_, where the number of ROS interactions per BSA molecule becomes insufficient to modify every molecule and the radiation-induced concentration effect is most pronounced, with ε is described by Function (4); (**III**) C_BSA_ ≫ αD, where the probability of BSA modification becomes very low and ε → 0%. Orange and blue curves correspond to doses D_1_ and D_2_, respectively, with D_1_ < D_2_ and C_tr__1_ < C_tr__2_. Red arrows indicate the direction of the e-beam irradiation. The distances d_1_, d_2_, and d_3_ represent the average intermolecular distances between BSA molecules at low, intermediate, and high protein concentrations, respectively (d_1_ > d_2_ > d_3_).

As it can be seen from Figure 8B,D, the dependencies ε(C,D) are determined by the ratio of the number of ionization events αD and the number of BSA molecules in the water solution and can be divided into three specific concentration ranges that are clearly reflected in the diagram (Figure 9).

In concentration range I, characterized by a low protein concentration, the maximum radiation-induced effect is observed. According to the Formula (4), if the concentration of BSA molecules C_BSA_ is significantly lower than the number of ionization events C_BSA_ ≪ αD, ensuring a sufficient amount of ROS for every BSA molecule to be modified, the ratio of radiation-modified proteins ε tends to 100%.

In the intermediate concentration range II, when the BSA concentration C_BSA_ exceeds the threshold concentration C_tr_, the number of BSA molecules starts growing above the number of ROS present in the water solution, which means that C_BSA_ > αD, so the number of ROS interactions per one BSA molecule becomes less than N_min_ required for the BSA modification effect to be detected. Such a ratio between the number of BSA molecules and the number of ROS interactions ensures the most pronounced radiation-induced effect on protein concentration, and that is where this radiation-induced BSA concentration effect is likely to occur.

In concentration range III, as the protein concentration increases, considering that the number of BSA molecules significantly exceeds the number of ROS, which means that C_BSA_ ≫ αD, the radiation-induced hyperbolic dependency ε(C,D) is proportional to αD/C_BSA_. In this case, the number of ROS interactions per one BSA molecule—much less than N_min_ required for the BSA modification effect to be detected—is deemed insufficient for detecting a radiation-induced BSA concentration effect due to a very low probability of damage to each BSA molecule, and with a further increase in the BSA concentration ε tends to 0%.

The difference in the rate of radiation-induced spatial BSA modification and peptide bond rupture in BSA molecules with an increase in the irradiation dose can be explained by the difference in the number of ROS interacting with one BSA molecule N_min_ required for spatial BSA modification or peptide bond rupture in BSA molecules to occur. Since the concentration dependency of the radiation-induced effect has the threshold concentration C_tr_, which is on the border between concentration ranges I and II, determining C_tr_ allows us to estimate the minimum interactions N_min_ of the BSA molecule with ROS required for the radiation-induced effect to occur.

To calculate C_tr_ we introduce a dimensionless variable x = αD/C. Then, the function (4) takes the form

(5) $$\varepsilon \left(x\right)=1-e^{-\frac{1}{x}}.$$

To quantify the concentration C_tr_, the minimum of the second derivative of the function (5) can be used, since it corresponds to the region of the maximum change in the curvature of the concentration dependency ε(C) at the fixed dose D (Figure 9):

(6) $$C_{tr}=min[\varepsilon^{\prime \prime }(C,D)].$$

The analysis of the second derivative of the function (5) shows that the minimum curvature is reached at x ≈ 0.21; thus, C_tr_ = 0.21·αD. The obtained value C_tr_ can be used as a border concentration between ranges I and II. If protein concentration C_BSA_ is less or equal to C_tr_, the number of ionization events is sufficient to affect all BSA molecules, and the observed radiation-induced effect is close to the maximum of 100%. If the protein concentration C_BSA_ is higher than C_tr_, the number of ionization events per one BSA molecule is less than N_min,_ which leads to a decrease in the ratio of the modified proteins in a water solution irradiated at the dose D.

Since the α coefficient determines the probability of BSA modification as a result of a single ionization event, in order to determine the N_min_ required for BSA spatial modification or peptide bond rupture in BSA molecules we need to determine α coefficients for these two different effects using approximation curves (4). According to the estimate, the coefficients α amount to (22.0 ± 0.2)·10^−4^ ((mg/mL)/Gy) and (1.75 ± 0.03)·10^−4^ ((mg/mL)/Gy) for the data obtained by the spectrophotometry method and the HPLC-MS/MS method, respectively. The given α coefficients can be recalculated in order to obtain the number of BSA molecules undergoing radiation-induced spatial modifications and peptide bond rupture per unit of the absorbed energy, which can be interpreted as the radiation-chemical yield (G-value) of the damaged BSA molecules. It has been established that the yield of the BSA molecules with the spatial radiation-induced modifications recorded using the spectrophotometry method is around 0.32 molecules/100 eV, while the yield of the BSA molecules in which peptide bond rupture was detected after trypsinolysis is around 0.025 molecules/100 eV.

### 3.3. Radiation-Chemical Interpretation of the Dose–Concentration Model

According to [56], the radiation-chemical yields of the key ROS occurring in water as a result of 1 MeV electron irradiation which would lead to chemical transformations of organic molecules are close in value to those occurring in water as a result of 7.5 MeV electron irradiation. The radiation-chemical yields for OH•, H•, H_2_, H_2_O_2_ and e_aq_^−^, calculated for 1 MeV electron irradiation, are 0.28, 0.062, 0.047, 0.073 and 0.28 μM/J, respectively [56,57]. The total ROS radiation-chemical yield can be estimated as

(7) $$G_{\Sigma }=0.28+0.062+0.047+0.073+0.28=0.742\;\frac{\mu M}{J}=0.742\;\frac{\mu M}{\mathrm{Gy}}.$$

Thus, the number of ROS occurring in 1 mL of water irradiated with 7.5 MeV electrons with the dose of 1 Gy can be calculated as

(8) $$N_{\Sigma }=0.742\cdot 10^{-6}\cdot \frac{6.022\cdot 10^{23}}{1000}=4.47\cdot 10^{14}\frac{ROS}{mL\cdot Gy}.$$

If protein concentration C_BSA_ is equal to C_tr_, this means that all BSA molecules interact with the number of ROS required for spatial BSA modification or peptide bond rupture in the BSA molecules to occur, N_min_. Thus, knowing the coefficients α and using the dependency of radiation-induced effect on the BSA concentration ε(C) in water irradiated at the fixed dose D allows us to calculate the threshold BSA concentration under irradiation at different doses using the formula C_tr_ = 0.21·αD. Knowing C_tr_ obtained for the BSA solution irradiated with the dose D and number of ROS $N_{\Sigma }$ found in 1 mL of water irradiated with 7.5 MeV electrons with the dose of 1 Gy allows us to estimate the minimum number of ionization events N_min_ required for a certain effect to be detected. It has been found that the minimum number of ROS interactions required for one BSA molecule to change its spatial modification is 112, while the peptide bond rupture that was detected after trypsinolysis require at least 1400 reactive oxygen species to interact with one BSA molecule. Therefore, to achieve a comparable effect, as recorded by HPLC-MS/MS, approximately 10 times more reactive oxygen species are required than for the appearance of detectable BSA spatial modifications involving partial denaturation, unfolding of BSA molecules and the formation of aggregates.

### 3.4. Practical Application of Dose–Concentration Model

During irradiation, high-protein food matrices and protein-based pharmaceuticals can undergo protein oxidation resulting in a change in protein conformation, destabilization of the tertiary structure formation of aggregates and other radiation-induced modifications. The use of our dose–concentration model (4) allows us to estimate the irradiation dose range which would be the most efficient for particular irradiation scenarios and products, bearing in mind the great diversity of proteins and their concentrations in food products of different categories and protein-based pharmaceuticals.

After conversion of the initial protein concentration C in the product to equivalent protein concentration in the aqueous extract C_conv_ and choice of the level of radiation-induced protein modifications ε which is permitted for the given product category, it is recommended to calculate the dose D at which the modifications in proteins in the product would not exceed the chosen level ε, using the following formula:

(9) $$D=-\frac{ln(\varepsilon /100\%)}{k(C)}.$$

Since bovine serum albumin is a stable and reliable reference point in studying the impact of physical and chemical factors on proteins, we have built our model around this protein. We recommend using the value k representing the rate of radiation-induced BSA modifications for the given initial BSA concentration C as the basis for estimating this value for other essential proteins contained in food matrices and protein-based pharmaceuticals in different concentrations to be able to identify irradiation dose ranges which would not cause the destruction of proteins and hence deteriorate the nutritional and functional value of products.

To summarize, a series of experiments performed using spectrophotometry and HPLCMS/MS analysis demonstrate that the dose dependency of radiation-induced BSA modifications in water solutions is particularly sensitive to BSA concentration. Our mathematical model factoring in the dose and concentration dependencies of the ratio of the radiation-induced BSA molecules has served as the basis for the computational model that knowing the initial protein concentration in a product can predict the degree of the damage to protein content when the product is irradiated at a specific dose. The degree of damage to the protein content in the product calculated for different irradiation doses can be used for determining the optimal irradiation dose range which would suppress pathogens in foods and protein-based pharmaceuticals while preventing functional or nutritional changes in the product. The proposed simplified model can be developed for use at industrial irradiation facilities since it allows us to calculate the optimal irradiation dose for the protein damage rate established for the given product with a particular protein content. However, considering that the current model does not take into account that real food and pharmaceutical matrices contain other solutes, such as sugars, lipids, antioxidants, enzymes, and buffers that can affect ROS scavenging and radiolysis pathways, thereby influencing protein damage rate, the next step of the research will involve studying the impact of food matrices and other factors on the damage rate of different proteins which can be found in foods and protein-based pharmaceuticals.

## 4. Materials and Methods

### 4.1. Objects of Study and Sample Preparation

Bovine serum albumin (BSA fraction V, BioClot Gmbh, Bavaria, Germany) in 0.9% NaCl solution at varying concentrations—0.5 mg/mL, 1 mg/mL, 5 mg/mL, 10 mg/mL, 20 mg/mL, 35 mg/mL and 70 mg/mL—was chosen as the object of the study (Stage 1 in Figure 1). Saline solution provides a reproducible saline medium with ionic strength relevant to biological and food systems while maintaining protein stability and solubility throughout the investigated concentration range. For irradiation, 0.5 mL aliquots of BSA solutions were placed in 2 mL sterile plastic microcentrifuge Eppendorf tubes without prior oxygen purging (Joint Stock Company Rybinsk Instrument-Making Plant, Rybinsk, Russia). Therefore, the samples contained both the dissolved oxygen initially present in the 0.9% NaCl solution and the air headspace enclosed within the tubes, allowing irradiation to proceed under aerobic conditions.

For the main irradiation series, 288 microcentrifuge tubes of BSA solutions with concentrations of 0.5 mg/mL, 1 mg/mL, 5 mg/mL, and 10 mg/mL were irradiated at six absorbed doses in the three successive irradiation sessions. For each dose and concentration, 12 tubes were used in total: 9 tubes for spectrophotometric analysis and 3 tubes for HPLC–MS/MS analysis taking into account the three-fold repetition. In addition, 24 tubes with BSA solutions at concentrations of 20 and 35 mg/mL were irradiated at 10 kGy.

Non-irradiated control samples were prepared separately: 12 tubes containing 0.5 mL BSA solutions at concentrations of 0.5 mg/mL, 1 mg/mL, 5 mg/mL and 10 mg/mL were used as controls for HPLC–MS/MS analysis, while 18 tubes containing 1.5 mL BSA solutions with concentrations ranging from 0.5 to 70 mg/mL were used as controls for spectrophotometric analysis.

### 4.2. E-Beam Irradiation

BSA solutions were irradiated using an ILU-14 pulsed electron accelerator (Budker Institute of Nuclear Physics of the Siberian Branch of the Russian Academy of Science, Novosibirsk, Russia) based at the Burnazyan Federal Medical Biophysical Center of FMBA of Russia (Moscow, Russia). The effective beam energy was 7.5 MeV and the beam power was 100 kW [58]. The width of the scanning field was 80 × 120 cm, the pulse repetition rate was 2–50 Hz, and the pulse current was 40–400 mA. During e-beam irradiation, BSA solution samples were placed at a distance of 50 cm from the beam output to ensure a higher dose uniformity (Stage 2 in Figure 1).

### 4.3. Dosimetry Control

The dose D absorbed by the BSA solutions and the dose rate during the irradiation were evaluated using a Fricke ferro-sulfate dosimeter. A 1.5 mL FeSO_4_ solution was placed in 2 mL microcentrifuge tubes and irradiated at the same parameters as the test samples. The density of the Fricke dosimetry solution is 1.025 g/mL and the density of the BSA solutions in a wide concentration range of 0.5–70 mg/mL varies from 1.005 to 1.023 g/mL; therefore, the doses absorbed by the dosimetry solution correspond to the doses absorbed by the BSA solutions.

The optical density of the Fricke dosimeter solution irradiated during an exposure time ranging from 10 to 100 s with a 10 s increment was measured at a wavelength of λ = 304 nm on a UV-3000 spectrophotometer (TM ECOVIEW, St. Petersburg, Russia) to estimate the dose rate absorbed by the BSA samples [59]. It was established that the dose rate absorbed by the Fricke solution irradiated with accelerated electrons generated at the given energy spectrum and beam parameters was 2.5 kGy/min. BSA samples were irradiated with the doses of 1, 2, 3, 5, 7, and 10 kGy. The ambient temperature during irradiation was 20 °C, and the control non-irradiated solutions were stored under the same temperature conditions as the irradiated solutions.

### 4.4. Spectrophotometry Analysis

The structural modifications in BSA as a result of irradiation were assessed using the spectrophotometry method (Stage 3 in Figure 1). Irradiated and non-irradiated solutions were placed in Ultra 10 mm quartz cuvettes with the dimensions of 12.5 × 12.5 × 45 mm^3^ (KU-10.10 A, Ultra Optic Cell Co., Ltd., Saint Petersburg, Russia). The solutions were stirred, and after 2–5 s the absorption spectra were measured using a UV-3000 spectrophotometer at the wavelengths ranging from 190 to 400 nm.

To quantify the ratio Q of radiation-modified BSA molecules, we used a coefficient calculated using the following formula:

(10) $$Q=\left(1-A_{D=0}/A_{D}\right)\cdot 100\%,$$

where A_D=0_ is the optical density of the non-irradiated BSA solution, and A_D_ is the optical density of the BSA solution irradiated with dose D measured at the wavelength of λ = 350 nm.

### 4.5. HPLC-MS/MS

The standards and equipment listed below were used to assess the structural integrity of the native BSA and to estimate its content.

Reagents

The following reagents were used for the experiment: bovine serum albumin standard, Fraction V (Bioclot GmbH, Lot No. 61171334, Aidenbach, Germany), formic acid (95%, Sigma-Aldrich, Cat. No. F0507, St. Louis, MO, USA), acetonitrile (HPLC-grade, AC03292500, Scharlau, Barcelona, Spain), sodium chloride (99%, Sigma-Aldrich, Cat. No. S9888, St. Louis, MO, USA), ammonium bicarbonate (99%, Sigma-Aldrich, Cat. No. A6141, St. Louis, MO, USA), and deionized water purified using a Milli-Q system (Millipore Merck, Cat. No. ZIQ7000T0C, Burlington, MA, USA). SMART Digest™ Trypsin Kits (Thermo Fisher Scientific, Cat. No. 60109-101, Waltham, MA, USA), used for enzymatic hydrolysis.

Consumables

The following consumables were used in the experiment: 10 kDa Amicon ultrafiltration filters (Cat. No. UFC801024, Millipore, St. Louis, MO, USA) and 30 kDa Amicon ultrafiltration filters (Cat. No. UFC503024, Millipore, St. Louis, MO, USA). Automatic pipettes 5–50 μL, 10–100 μL, 20–200 μL, and 100–1000 μL (Labmate, Chicago, IL, USA) with a maximum measurement error of ±5% were used for accurate aliquoting. Reagents were weighed using Vibra analytical balances with a readability of 0.0001 g (SHINKO DENSHI, Tokyo, Japan).

Equipment

To analyze the degradation of the BSA solutions and quantify the peptide content as a function of exposure dose, we used an Ultimate 3000 RSLC liquid chromatograph (Dionex, Germering, Germany) with an automatic sample-introduction system and an Orbitrap Fusion Lumos high-resolution mass spectrometer (Thermo Fisher Scientific, Waltham, MA, USA) containing an electrospray ionization source. Chromatographic separation was performed on a Zorbax 300 SB-C18 HPLC column (100 mm × 2.1 mm, sorbent grain diameter 3.5 μm) manufactured by Agilent Technologies (Santa Clara, CA, USA). Chromatograms were recorded using Xcalibur version 1.5 (Thermo Fisher Scientific, Waltham, MA, USA) software packages.

Sample Preparation

An aliquot of 30 µL of the irradiated and non-irradiated BSA solutions containing 0.9% NaCl was mixed with 210 µL of 1 M NH_4_HCO_3_ and transferred to a 0.5 mL Amicon centrifugal filter with a 30 kDa molecular-weight cutoff. Prior to use, the filter unit was pre-rinsed twice with 500 µL of deionized water (15 min at 10,000 rpm each). The resulting 50 µL protein concentrate was recovered by ultracentrifugation for 3 min at 1000 rpm into a 2 mL microcentrifuge Eppendorf tube.

The concentrate was supplemented with 12 µL of 1 M NH_4_HCO_3_ and 90 µL of the digestion buffer supplied with the SMART Digest™ Trypsin Kit. After homogenization, 3 µL of a 1 mg/mL trypsin solution was added, and the mixture was vortexed thoroughly. Protein digestion was carried out at 70 °C for 2 h, followed by brief vortexing.

The digestion mixture was subsequently transferred to a 0.5 mL 10 kDa Amicon centrifugal filter and centrifuged at 2000 rpm for 10 min. The filtrate was collected into a micro vial and subjected to HPLC–MS/MS analysis.

HPLC-MS/MS analysis

The electrospray ionization source was used in the mode of registration of positively charged ions. The resolution of the mass analyzer was not less than 30,000 rel.un. and the error in determining *m*/*z* values did not exceed 3 ppm. Chromatographic separation was carried out in gradient elution mode. The mobile phase A was 0.1% formic acid in water, and the mobile phase B was acetonitrile. All the conditions of HPLC-MS analysis are summarized in Table A2 in Appendix A.

Selected Peptides

To assess the effect of accelerated electrons on the structural characteristics of bovine serum albumin, three peptides—FKDLGEEHFK (T35–44), AEFVEVTK (T249–256), and KQTALVELLK (T548–557)—were selected and employed for this purpose in the study [60]. The chromatography and mass-spectrometry characteristics of these peptides are presented in Table 1.

### 4.6. Statistical Analysis of the Data

The BSA solutions at different concentrations were irradiated with accelerated electrons three times at each irradiation dose. For each non-irradiated and irradiated solution, the absorption spectra were measured three times in order to estimate the margin of error in the determining optical density at the critical wavelength.

Statistical processing and the plotting of all approximating curves were performed using the Origin Pro 2024 10.1.0.178 program (OriginLab Corporation, Northampton, MA, USA). All graphs present experimental data as means ± SD format.

## 5. Conclusions

The study involving 7.5 MeV e-beam irradiation of bovine serum albumin (BSA) solutions with doses ranging from 1 kGy to 10 kGy has revealed that the degree of radiation-induced conformational changes in the BSA molecules registered using the spectrophotometry method and the peptide bond rupture in the BSA native structure detected after trypsinolysis using HPLC-MS/MS is not only strictly dose-dependent but also is largely determined by the initial protein concentration. For the entire range of BSA concentrations from 0.5 mg/mL to 70 mg/mL, an increase in the ratio of the radiation-induced modifications in the BSA molecules was observed with an increase in the radiation dose, while an increase in the BSA concentration was accompanied by a decrease in the rate of radiation-induced changes. Such an effect of the initial BSA concentration on the degree of radiation-induced conformational changes and peptide bond rupture in the BSA native structure allows us to determine a minimum number of interactions of reactive oxygen species (ROS) with one BSA molecule required for each BSA modification. It has been estimated that a ten-fold larger number of ROS interactions with BSA molecules is required for the destruction of BSA native structure compared to the number of ROS interactions leading to significant conformational changes in proteins. The combination of spectrophotometry and liquid chromatography coupled with mass spectrometry has revealed that e-beam irradiation-induced changes in proteins follow a specific pattern: conformational rearrangements that prevail immediately after exposure to e-beam irradiation are succeeded by irreversible oxidative modifications accompanied by aggregation and peptide bond rupture in the native structure of proteins.

The proposed dose–concentration model factoring in the aforementioned findings allows us to quantify the degree of radiation-induced protein modifications depending on the irradiation dose and protein concentration in model protein suspensions. For use at industrial irradiation facilities, this model should be refined to ensure a higher precision of the optimal dose estimate for real food and pharmaceutical matrices that are not limited to albumins but also contain other components which can influence the rate of protein damage. A comprehensive approach which regards each product as a complex system of proteins, fats and hydrocarbons with different radiosensitivities, whose damage depends not only on the type of biopolymers but also on their initial concentration, will allow us to assess the stability of components responsible for the nutritional value of the product in order to determine scientifically based irradiation regimes for e-beam industrial food irradiation. Furthermore, we are going to extend the current research by studying the impact of irradiation on proteins in real food matrices with different protein contents and other food components which should be preserved during irradiation.

## Figures and Tables

**Table 1:** Characteristics of the selected BSA peptides used to evaluate the effect of accelerated electrons on the structural properties of bovine serum albumin [60].

Peptide	Exact *m*/*z* Value of the Precursor Ion	Amino Acid Sequence of the Precursor	Selected Ion Transitions	t_R_, min
T35–44	417.2119	FKDLGEEHFK	1. *m*/*z* 417.21 → *m*/*z* 746.3468 2. *m*/*z* 417.21 → *m*/*z* 294.1812	11.4 ± 0.2
T548–557	571.8608	KQTALVELLK	1. *m*/*z* 571.86 → *m*/*z* 1014.6202 2. *m*/*z* 571.86 → *m*/*z* 886.5608	17.8 ± 0.2
T249–256	461.7477	AEFVEVTK	1. *m*/*z* 461.75 → *m*/*z* 722.4084 2. *m*/*z* 461.75 → *m*/*z* 476.2715	11.6 ± 0.2

## Data Availability

The original contributions presented in the study are included in the article; further inquiries can be directed to the corresponding author.

## Appendix A

**Table A1:** Bovine serum albumin content in samples of different concentrations after 7.5 MeV electron beam irradiation (n = 3).

Concentration, mg/mL	Peptide	C_Peptide_, %					
		1 kGy	2 kGy	3 kGy	5 kGy	7 kGy	10 kGy
0.5	T35–44	62.0 ± 4.5	33.4 ± 2.9	12.3 ± 1.5	7.5 ± 1.1	1.53 ± 0.31	1.05 ± 0.06
	T402–412	65.2 ± 4.2	38.2 ± 2.7	24.1 ± 2.0	16.9 ± 1.9	6.7 ± 0.8	5.4 ± 0.2
	T548–556	79.7 ± 9.9	46.3 ± 6.4	23.6 ± 2.1	12.9 ± 2.2	4.1 ± 1.1	2.3 ± 0.3
1	T35–44	81.5 ± 0.8	65.5 ± 1.5	69.5 ± 1.7	22.3 ± 0.5	15.2 ± 0.4	7.5 ± 0.3
	T402–412	79.9 ± 0.6	68.6 ± 1.6	71.2 ± 2.0	39.8 ± 1.1	33.3 ± 0.8	21.8 ± 0.7
	T548–556	88.4 ± 1.4	79.7 ± 2.2	78.5 ± 1.4	35.5 ± 1.6	28.9 ± 1.7	16.8 ± 1.0
5	T35–44	100.9 ± 1.6	98.8 ± 0.6	87.2 ± 0.9	68.8 ± 0.9	60.0 ± 1.1	41.6 ± 0.8
	T402–412	94.7 ± 2.3	92.2 ± 2.0	87.7 ± 1.8	74.8 ± 1.4	70.8 ± 1.7	61.5 ± 1.7
	T548–556	100.0 ± 0.9	99.1 ± 0.2	93.4 ± 0.6	82.5 ± 0.5	78.6 ± 0.5	69.1 ± 0.6
10	T35–44	86.6 ± 1.4	87.7 ± 1.3	83.6 ± 1.7	80.9 ± 1.3	85.1 ± 0.6	83.6 ± 2.1
	T402–412	101 ± 5	91.7 ± 6.2	94.8 ± 5.9	93.9 ± 4.5	89.0 ± 5.7	77.3 ± 5.2
	T548–556	101 ± 2	93.4 ± 1.3	98.9 ± 2.1	97.8 ± 0.8	94.0 ± 0.8	91.4 ± 0.6

**Table A2:** HPLC-MS/MS analysis conditions.

HPLC-MS/MS Parameters	Value
	HPLC
Column	Zorbax 300 SB-C18 HPLC column (100 mm × 2.1 mm, sorbent grain diameter 3.5 μm)
Mobile phase	Mobile phase A: 0.1% formic acid in water
	Mobile phase B: 100% acetonitrile
Flow rate	0.3 mL/min
Column temperature	40 °C
Injection volume	10 µL
Gradient elution mode	0–3 min—95% A; 3–35 min—5–40% B; 35–40 min—40–80% B; 40–44 min—80% B; 44–50 min—95% A.
	MS/MS
Ionization method	Electrospray ionization (ESI)
Ion mode	Positive polarity
Ion source temperature	325 °C
Drying gas pressure	420 kPa
Nebulizing gas pressure	70 kPa
Capillary voltage	3500 V
Mass scanning mode	Selected reaction monitoring (see Table 1)
